# 

**Published:** 1864-05

**Authors:** 


### THE CHICAGO

### MEDICAL JOURNAL

DE LASKIE MILLER, M. D.,
EPHRAIM INGALS, M. D.,
Editors and Proprietors.

MAY, 1864.

CHICAGO:

J. C. W. BAILEY, BOOK AND JOB PRINTER, 130 CLARK STREET.

1864.

